# Effect of warming eyelids on tear film stability and quality of life in visual display terminal users: a randomized controlled trial

**DOI:** 10.1038/s41598-020-73779-6

**Published:** 2020-10-09

**Authors:** Chi-Chin Sun, Chia-Yi Lee, Yih-Shiou Hwang, Igaki Michihito, Kyoko Tagami, Ching-Hsi Hsiao

**Affiliations:** 1 Department of Ophthalmology, Chang Gung Memorial Hospital, Keelung, Taiwan; 2 Department of Medicine, School of Medicine, Chang Gung University, Taoyüan, Taiwan; 3 Department of Ophthalmology, Show Chwan Memorial Hospital, Changhua, Taiwan; 4 Department of Ophthalmology, Chang Gung Memorial Hospital, Linkou, Taiwan; 5 Personal Health Care Products Research Laboratories, Kao Corporation, Sumida-ku, Tokyo, Japan

**Keywords:** Conjunctival diseases, Corneal diseases, Lacrimal apparatus diseases

## Abstract

We aimed to evaluate the effect of warming eyelids on tear-film stability and quality of life (QoL) in video display terminal (VDT) users. A prospective study was conducted and 45 volunteers with ocular symptoms and tear-film instability associated with VDT use were randomly allocated into the study (n = 22) or control groups (n = 23). Subjects in the study group used eyelid warming steamer (EWS) for 2 weeks and tear fluorescein breakup time (TBUT) after single and 2-week EWS treatment, Schirmer I test, ocular surface staining scores, meibomian gland assessment, severity of dry eye disease (DED) and QoL scores after 2-week EWS treatment were analysed. The TBUT improved after both single and 2-week EWS treatment (*P* = 0.023 and 0.027, respectively) in the study group. The ocular surface staining scores were significantly decreased only in the study group (*P* = 0.038). About 60% DED patients in the study group shifted towards non-DED and the pattern of distribution was significantly different compared to baseline (*P* < 0.001). The QoL scores significantly improved in the study group (*P* = 0.002) with a negative correlation with TBUT. In conclusion, in VDT users with short TBUT, eyelid warming steamer is effective in improving tear-film stability and QoL.

## Introduction

As the development and utilization of electrical device, visual display terminal (VDT) has become essential equipment in the current life^1^. According to an estimation, there are one billion personal computers throughout the world^2^, which even does not include the cell phones and monitors. Consequently, several complications such as musculoskeletal and dermatological disorders, psycological stress, and ocular problems have been reported in those VDT users^3–8^.

Several ocular complications including comitant esotropia, accommodation defect, and dry eye disease (DED) have been found to correlate with VDT user^9–12^. The meta-analysis reported the global prevalence of DED in VDT users ranging from 9.5 to 87.5% based on variable diagnostic criteria^12^. According to the Osaka study, a large-scale study conducted in Japan, the prevalence of DED (including definite and probable dry eye based on Japanese Dry Eye Criteria) in VDT users was 76.5% and 60.2% in women and men, respectively, and ocular findings included short tear breakup time (TBUT) and corneal staining accompanied by normal Schirmer test values; women older than 30 years old and prolonged VDT user increased risk for DED^13^. The subsequent Osaka report indicated DED had a significant impact on work productivity loss^14^. Meibomian gland dysfunction (MGD), a main cause for evaporative dry eye, was also prevalent in VDT users, and MGD parameters were correlated with VDT working time^15^. Since VDT users is a growing population, VDT related ocular surface disorder will become an important issue on public health. Thus, measures to decrease the adverse impact on ocular surface in VDT users cannot be overlooked.

The eyelid warming device with steam has been used to relieve the degree of MGD by melting the meibomian gland lipid from the early 2000s^16,17^. Compared to warmed facecloth, the eyelid warming masks had been proven to retain heat more effectively^18^, and the tear evaporation rate, TBUT and Schirmer test value were improved in individuals with MGD after the application of lid warming steam instrument^19,20^. Recently, Arita et al. reported that the DED-related indexes such as tear meniscus volume and TBUT significantly increased via the use of disposable eyelid-warming steamers in DED patients^21^. Thus, it seems reasonable to apply eyelid warming steamer device to VDT users which has not been evaluated before.

Hence, we conducted a prospective, randomized study to evaluate the effect of eyelid warming steamer on tear film stability and QoL in VDT users with short TBUT.

## Results

### Demography of the study population

Forty-five subjects (10 males and 35 females: age 36.2 ± 7.9 years old) with eye symptoms and short-TBUT associated with the prolonged VDT use were enrolled in this study and they randomly allocated into the study group (n = 22) or the control group (n = 23). The baseline characteristics and ocular findings of the subjects in each group are shown in Table 1. There were no significant differences in the baseline characteristics of ocular findings between two groups.

**Table 1:** Baseline characteristics and ocular findings of the subjects in each group.

	Study group (n = 22)	Control group (n = 23)	P value
Age (year, mean ± SD)	35.6 ± 6.3	36.7 ± 9.4	0.820
TBUT (sec, mean ± SD)	3.0 ± 1.1	2.6 ± 1.3	0.232
**Schirmer test (mm, Mean ± SD)**	14.0 ± 11.7	10.8 ± 9.1	0.260
> 5 mm	17 (77.2%)	13 (56.5%)	
≤ 5 mm	5 (22.7%)	10 (43.5%)	
**Ocular damage score (mean ± SD)**	1.7 ± 1.7	1.8 ± 2.2	0.527
0–2	17 (77.3%)	18 (72.0%)	
3–6	5 (22.7%)	5 (20.0%)	
7–9	0 (0.0%)	2 (8.0%)	
**Meibum quality and expressibility score (mean ± SD)**	1.5 ± 0.7	1.3 ± 0.5	0.434
Grade 0	0	1 (4.0%)	
Grade 1	14 (63.6%)	16 (52.0%)	
Grade 2	6 (27.3%)	8 (36.0%)	
Grade 3	2 (9.1%)	0 (0.0%)	
**Meibomian gland dropout score (mean ± SD)**	0.6 ± 0.6	0.7 ± 0.6	0.699
Grade 0	10 (45.5%)	9 (39.1%)	
Grade 1	11 (50.0%)	13 (56.5%)	
Grade 2	1 (4.5%)	1 (4.4%)	
Grade 3	0 (0.0%)	0 (0.0%)	

*SD* standard deviation.

### Dry eye parameters after the management

**Figure 1:**
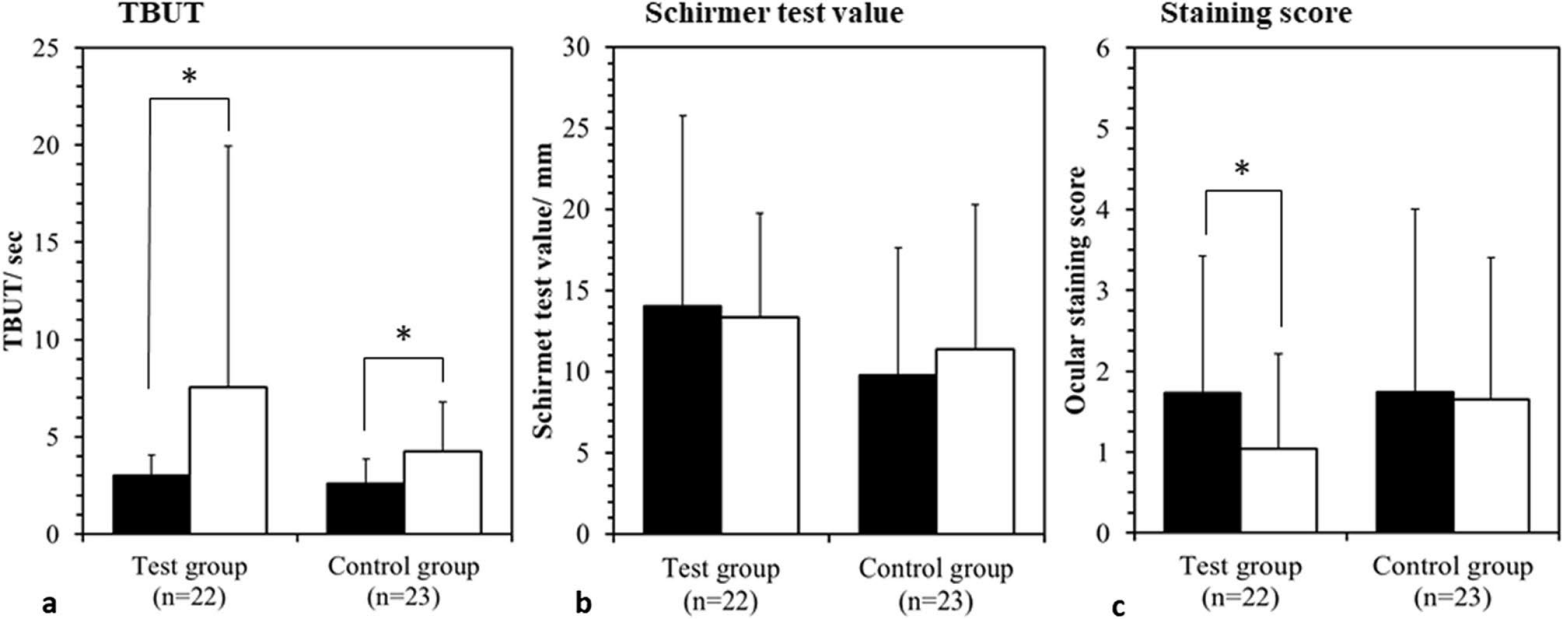
Change in dry eye parameters at the baseline (black column), after 2-week treatment (white column) in the test group and the control group. (**a**) The change in TBUT. (**b**) The change in Schirmer test value. (**c**) The change in ocular surface staining score. *Indicates the significant difference between the baseline and after 2-week treatment with *P* < 0.05.

TBUT was measured before and after 10-min single treatment with EWS or non-warming eye mask as the immediate effect of each treatment on tear film instability. While there was no significant difference in TBUT with the single treatment with non-warming eye mask in the control group (before: 2.6 ± 1.4 s, after: 3.4 ± 2.6 s), TBUT was significantly increased from 3.0 ± 1.1 s to 4.1 ± 2.5 s with the single treatment of EWS in the study group (*P* = 0.023).The changes in TBUT, Schirmer test value and ocular surface staining scores with 2-week treatment are illustrated in Fig. 1. Compared to the baseline, TBUT was significantly increased with 2-week treatment with EWS in the study group (*P* = 0.027) as well as with non-warming eye mask in the control group (*P* = 0.003). The ocular surface staining scores were significantly decreased in the study group (*P* = 0.038), however, no significant difference was found in the control group. The Schirmer test value did not change significantly in both groups.

### Meibomian gland score between the groups

To investigate the effect of lipid secretion from meibomian glands on the improvement of tear film stability, the change in meibomian gland dropout scores and meibum expressibility scores in the subjects with increased TBUT with 2-week treatment in the study group (n = 11) and the control group (n = 15) were analyzed. The results of meibomian gland dropout scores and meibum expressibility scores are presented in Table 2. There was no change in the meibomian gland dropout scores with 2-week treatment in both groups, however, meibum expressibility scores tended to be improved in the study group (*P* = 0.054), while the scores did not significantly change in the control group.

**Table 2:** Change in the meibomian gland dropout scores and meibum expressibility scores in the subjects after 2-week treatment.

	Study group (n = 11)		Control group (n = 15)	
	Baseline	2-week treatment	Baseline	2-week treatment
**Meibomian gland dropout scores**				
Grade 0	5 (45.5%)	5 (45.5%)	7 (46.7%)	7 (46.7%)
Grade 1	6 (54.5%)	6 (54.5%)	7 (46.7%)	7 (46.7%)
Grade 2	0 (0.0%)	0 (0.0%)	1 (6.6%)	1 (6.6%)
Grade 3	0 (0.0%)	0 (0.0%)	0 (0.0%)	0 (0.0%)
Mean ± SD	0.6 ± 0.5	0.6 ± 0.5	0.6 ± 0.6	0.6 ± 0.6
*P* value		1.000		1.000
**Meibum expressibility scores**				
Grade 0	0 (0.0%)	3 (27.3%)	1 (6.7%)	0 (0.0%)
Grade 1	6 (54.5%)	6 (54.5%)	9 (60.0%)	10 (66.7%)
Grade 2	4 (36.4%)	2 (19.2%)	5 (33.3%)	4 (26.7%)
Grade 3	1 (9.1%)	0 (0.0%)	0 (0.0%)	1 (6.7%)
Mean ± SD	1.6 ± 0.7	0.9 ± 0.7	1.3 ± 0.6	1.5 ± 0.6
*P* value		0.054		0.487

### Alteration of dry eye degree distribution

The change in the distribution of three diagnostic DED categories was analyzed (Table 3). At the baseline, 22.7% and 26.1% of the subjects were diagnosed as definite DED in the study group and the control group respectively and the rest of the subjects were diagnosed as probable DED. After 2-week treatment, 59.1% of the subjects who were diagnosed as definite DED or probable DED at the baseline were diagnosed as non-DED and the distribution of DED categories was significantly shifted towards non-DED in the study group (*P* = 0.000). On the other hand, while 21.7% of the subjects with definite DED or probable DED at the baseline were improved to non-DED after 2 weeks in the control group, the distribution after 2 weeks was not significantly improved with the comparison to the baseline, and the distribution in the control group after 2 weeks was significantly worse than in the study group (*P* = 0.038).

**Table 3:** Change in the distribution of diagnostic dry eye disease categories by Japanese dry eye disease criteria.

	Study group (n = 22)			Control group (n = 23)		
	Baseline	2-week treatment	*P* value	Baseline	2-week treatment	*P* value
Definite DED (%)	5 (22.7%)	2 (9.1%)	0.000^b^	6 (26.1%)	4 (17.4%)	0.058
Probable DED (%)	17 (77.3%)	7 (31.8%)		17 (73.9%)	14 (60.9%)	
Non-DED (%)^a^	0 (0.0%)	13 (59.1%)		0 (0.0%)	5 (21.7%)	

^a^Denote the comparison between the two groups, the baseline distribution between the two groups was identical (*P* = 0.793) while the study group significantly switched to non-DED status compared to the control group after 2-week treatment (*P* = 0.038).

^b^Denote significant differences between baseline and after 2-week treatment in each group (P < 0.05).

### Subjective symptoms and quality of life

**Figure 2:**
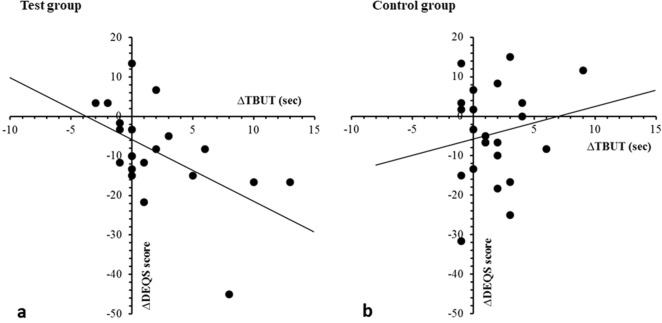
Correlation between the change in tear fluorescein breakup time and Dry Eye-Related Quality of Life Score. (**a**) The result in the test group. (**b**) The result in the control group.

Regarding to the effect of warming eyelids on QoL in the subjects with tear film instability associated with the prolonged VDT use, DEQS score as the parameter of QoL was significantly improved after 2-week treatment in the study group (baseline: 28.5 ± 14.0, after 2-week treatment: 19.8 ± 12.9, *P* = 0.002), however, there was no significant difference in DEQS between at the baseline and after 2 weeks in the control group (baseline: 30.3 ± 14.0, after 2-week treatment: 25.9 ± 15.6). The correlation between the improvement of TBUT and DEQS score in each group is shown in Fig. 2. There was a moderate correlation between increased TBUT and decreased QoL impairment in the study group (correlation coefficient, *r* = 0.540), while no significant correlation was found in the control group.

## Discussion

High prevalence of DED and MGD has been reported in VDT users^12,15,22,23^. The mechanism of VDT use related DED and MGD is not very clear. In VDT users, it is reported that spontaneous blinking rate decreases, and incomplete blinking increases^24^, which may cause rapid evaporation of tear film, then lead to tear film instability, ocular surface inflammation and the following DED. In addition, the muscular action during blinking is one of driving forces to deliver meibum. With incomplete blinking, meibum expression may reduce, which may lead to stasis of meibum, then gland dropout finally. In the current study, the effectiveness of EWS has been well-demonstrated. After the 2-week application, the QoL, TBUT, ocular surface staining scores and distribution of DED categories significantly improved in the study group compared to the control group.

Common parameters to evaluate the tear film stability include TBUT, Schirmer test and ocular surface staining scores. In the current study, the TBUT significantly improved just after the single application of EWS. Arita et al. also found that TBUT improved after application of eyelid warming steamer containing menthol in healthy subjects and patients with dry eye^21^; which may imply that even single use of warm steamer is sufficient to prolong TBUT. It is not surprizing that the value of Schirmer test did not significantly improve in the current study, because DED in VDT users featured with a shortened TBUT, but a normal value of Schirmer test according to Osaka study^13^, which suggested the aqueous deficiency may not be the major defect of DED in VDT users. In addition, TBUT has become a more reliable ocular surface stability test for DED according to the latest consensus of Asia Dry Eye Society^25^, and the prevalence of evaporative type of DED was much more than the aqueous tear deficiency type of DED^26^. In current study, for the first time, we found that corneal and conjunctival vital staining scores were significantly reduced by 2-week application of EWS in VDT users (Fig. 1).

It is well known that warm compression would improve the meibum quality and meibum expressibility^27^ and increase meibomian gland area in the patients with MGD^20^. Although MGD is one of major ocular disorders in the VDT users^13^, little work has evaluated MG changes after warm compression in VDT users. In our study, the meibum expressibility tended to improve, but the meibomian gland dropout scores remained unchanged after 2-week therapy. In previous studies about the effect of warm compression on MGD patients, the treatment period was 1 month^20,27^, while the treatment interval was only 2 weeks in the current study. Moreover, additionally mechanical meibomian gland squeezing plus eyelid scrubs were performed by Lee et al.^27^. Whether a longer treatment period would significantly improve the therapeutic effect deserves further investigation. Also, less than 10% of patients in the current study belonged to grade 3 meibum expressibility, so limited improvement may occur in VDT users while compared to those with severe MGD^20,27^.

Apart from eyelid warming, other interventions for VDT-related ocular disorders have been reported. Taking bilberry extract for 8 weeks and Omega 3 fatty acid for 45 days have been shown to improve clinical symptoms, tear stability, and conjunctival cytology in symptomatic VDT users^28,29^. In addition, moist cool air device, a special equipment designed for office use, had also been applied four hours per day for 5 days to VDT users, then improvement of dryness, functional visual acuity and tear film stability was found^30^. Compared with these treatments, the eyelid warming program in our study is fast, simple, convenient, because the treatment takes 15 min per day, and had TBUT prolonged after single application and various tear film indexes improved after 2-week application.

It is evident that in VDT users, most DED individuals presented with prominent ocular symptoms and short TBUT^25^. However, scanty research evaluates the effectiveness of eyelids warming on alternating DED severity. After the 2 weeks treatment, the severity of DED in the study group was significantly reduced with 59.1% of the definite DED or probable DED patients re-diagnosed as non-DED condition while no change was found in the control group. Since no patient was diagnosed with non-DED in both the study group and the control group initially, the significant change in the study group is encouraging that more than half of VDT users can be recovered from DED by the utilization of EWS device. Interestingly, approximately one-fifth of participants in the control group were diagnosed with non-DED after the follow-up period. Reviewing the diagnosis criteria of definite-DED in this study, there are two possible explanations for the improvement of DED in no-treatment participants. First, the non-warming eye mask may have certain placebo effect on those patients in the control group thus the subjective eye symptoms could be “psychologically” relieved. On the other hand, the self-awareness toward DED may arise after the enrollment into the study and patients in the control group put some extraordinary effects to prevent DED progression which reflects on the improvement of TBUT and non-significant increment of Schirmer’s test (Fig. 1). Overall, although VDT users would develop severe DED, the severity and distribution of DED in the VDT users can be simply reversed by EWS device considering the subjective and objective parameters of DED.

Since the QoL of VDT users were reduced^31^, the QoL should also be regarded as an index of treatment effect in the VDT users. In this study, the QoL evaluated by DEQS scores were both decreased in the study group and the control group but only the decrement in the study group was significant. Similar with the trend from DED to non-DED, the DEQS score was also decreased in the control group which may also be resulted from the placebo effect. Moreover, the moderate correlation between increased TBUT and decreased QoL impairment implying that TBUT could not only serve as an important test of tear film instability but also could be used to evaluate the QoL in VDT users.

There are several limitations in this study. First, the small study population may limit statistical significance. Second, the 2-week treatment interval may be not long enough for a complete evaluation. Furthermore, since each subject can find each device is warm version or not, this study cannot be set as a double-blind study. Moreover, the female predominance with a ratio more than 4:1 in the current study may not represent the general population. However, the prospective nature and the randomized-controlled design of the current study can provide strong evidence about the treatment effect with minimal bias.

In conclusion, the eyelid warming management with the EWS can significantly improve the TBUT, ocular surface staining scores, distribution of DED status and the QoL in VDT users with short TBUT. Although meibum expressibility score remained unchanged after the eyelid warming management, a trend toward statistical improvement had been observed. Further large-scale study with longer follow-up interval to validate the optimal protocol of eyelid warming for VDT users to maintain a stable tear film condition is mandatory.

## Methods

### Ethics declaration

The study protocol was approved by the Institutional Review Board of Chang Gung Memorial Hospital (I04-8007B). This study adhered to the tenets of the Declaration of Helsinki, in compliance with the International Conference on Harmonisation Good Clinical Practice and local laws and regulatory requirements (Supplementary Information).

### Study design and subjects

A 2-week randomized controlled trial was conducted from January 2016 to March 2016. The study coordinator fully explained the nature of the procedures to all subjects and they signed written informed consents prior to enrolment. We recruited the volunteers with any eye symptoms through a dry eye questionnaire as well as tear film instability associated with the prolonged VDT use in a clinic (Day 0). Briefly, we included the subjects using visual display terminals (VDTs) for 6 h or more a day over 3 months, with at least one of 12 persistent eye symptoms that were experienced “often” or “constantly” in a dry eye questionnaire developed by Toda et al.^32^, and with decreased TBUT ≤ 5 s. We excluded the subjects with the excessive meibomian lipid secretion (seborrheic MGD), eye abnormalities (e.g. infectious conjunctivitis, allergic disease, autoimmune disease or collagen disease), skin abnormalities around eyes (e.g. trauma, rash, swelling or eczema) or any history of hypersensitivity or diminished sensitivity to the heating. The eligible subjects were randomly allocated into a study group or a control group with the assessors blinded and visited the clinic a week (Day 1) and three weeks after (Day 15) with the interval of 2-week test period to examine ocular condition. They visited the clinic at the same time period for each visit. The contact lenses users were required to take them off before visiting the clinic.

### Treatment with Eyelid Warming Steamer (EWS)

Eyelid Warming Steamer (EWS) is an eye mask in shape, which contained iron (Fe) and water in the sealed heating unit and was designed to generate heat with steam (moist heat) by oxidative reaction of iron. EWS was individually packed in a pouch, and once the pouch was opened, EWS immediately began to warm up with the oxidative reaction initiated. The temperature of generated moist heat was approximately 40 °C and it lasted for about 15 min. The chemical reaction automatically terminated in about 15 min and was unavailable to reuse. The subjects in the study group used EWS for 10 min in the clinic at Day 1 to evaluate the short-term effect of the single treatment on ocular condition and they used EWS once a day for 2 weeks at home. They visited the clinic at Day 15 to evaluate the long-term effect with the 2-week treatment. The subjects in the control group were required to use a non-warming eye mask that had the same shape appearance as EWS and did not generate the heat and the steam. The usage condition of a non-warming eye mask was totally the same as EWS.

### Outcome measures

#### Eye examinations

Tear function and ocular surface condition were evaluated by corneal and conjunctival vital staining test, TBUT test and Schirmer I test as tear film stability was set as a primary outcome in eye examinations. The corneal and conjunctival epithelial damages were evaluated by the single staining method of fluorescein dye with a yellow barrier filter observation. After the liquid of fluorescein was instilled by the micropipette to stain ocular surface, subjects were asked to blink several times to mix fluorescein with tears. The ophthalmologist observed the epithelial damage in three areas segmented into cornea, nasal conjunctiva and temporal conjunctiva and scored from 0 (absence) to 3 (widespread loss of epithelium) for each area^33^. The total score for corneal and conjunctival vital staining ranged from 0 to 9. TBUT test was conducted with slit lamp observation to tear film stained by fluorescein dye to evaluate tear film stability. After dye instillation, subjects were also asked to blink several times and keep eye open without blinking. The time interval between the last complete blink and the first dark spot observed on the corneal surface was measured by a stopwatch. Schirmer I test was performed without topical anesthesia to evaluate tear flow reflex and quantity. A Schirmer paper strips was placed at lower lid margin at the outer one third portion. Subjects were requested to close their eyes for 5 min and the strip was removed and recorded the length of wet portion in the strip. To avoid the influence on tear function and ocular surface condition, Schirmer I test was conducted at the end of the procedures at each visit.

Meibomian glands were also assessed to evaluate the effect of lipid secretion from meibomian glands on tear function and ocular surface condition. The meibum quality and expressibility at upper eyelid was evaluated with a physical force applied to the outer surface of the eyelid by scoring from 0 to 3 as follows: grade 0, clear meibum and easily expressed; grade 1, cloudy meibum and easily expressed; grade 2, cloudy meibum expressed with moderate pressure; and grade 3, meibum not expressible even with hard pressure^34^. The meibomian gland dropout at upper eyelid was observed using Karatograph 5 M (Oculus, Wetzlar, Germany) and scored from 0 to 3 as follows: grade 0, no loss of meibomian glands; grade 1, lost area was less than one third of the total area of meibomian glands; grade 2, lost area was between one third and two thirds of the total area of meibomian glands; and grade 3, lost area was over two thirds of the total area of meibomian glands^35^.

To clarify the short-term effect of the treatment on tear film instability as a primary outcome, TBUT test was conducted before and after the single treatment at Day 1. Also, all eye examinations described above were performed at the baseline at Day 1 and after 2-week treatment at Day 15 to reveal the long-term effect.

#### Diagnosis of DED

According to the Japanese dry eye diagnostic criteria^36^, DED was diagnosed based on the results of the questionnaire and eye examinations with 2-week treatment in the current study. This diagnosis included three criteria as follows: (1) presence of eye symptoms (at least one of 12 eye symptoms that experienced “often” or “constantly”), (2) presence of qualitative or quantitative tear film abnormality in 1 or both eyes (TBUT ≤ 5 s or Schirmer I test ≤ 5 mm) and (3) presence of corneal and conjunctival epithelial damage (total staining score ≥ 3 points out of 9 points). The subjects corresponding to all 3 criteria were diagnosed as definite DED, and those corresponding to 2 of the 3 criteria were diagnosed as probable DED, while those corresponding to 1 or no positive criteria were diagnosed as non-DED.

### Questionnaire on eye symptoms and QoL

We administrated a dry eye questionnaire that was also used at the screening procedures to assess the subjective eye symptoms. This questionnaire was widely used for the assessment of ocular surface-related eye symptoms and includes 12 questions on the frequency of the perception of eye symptoms with 4 possible answers; “never”, “sometimes”, “often” and “constantly”. To evaluate the effect of eye symptoms and ocular condition on quality of life (QoL), ocular surface-related QoL was assessed using Dry Eye-Related Quality of Life Score (DEQS) developed by the Japanese Dry Eye Society^37^. This questionnaire consists of 15 questions related to dry eye symptoms and influence on daily life, and the overall degree of QoL impairment is calculated as a summary score, 0 to 100. Both questionnaires were completed at Day 1 and Day 15 by each subject prior to eye examinations.

### Statistical analysis

All data were indicated as means ± standard deviation (SD). The comparison between before and after single or 2-week treatment in each group was conducted using paired *t*-test for parametric variables and Wilcoxon singed-rank test for non-parametric variables. The comparison between two groups was performed using Mann–Whitney U test. The chi-squared test was used for the comparison of the variable distribution between two groups. P value < 0.05 was considered as statistically significant with a confidential interval of 95%. All these statistical analyses were conducted using SPSS software (IBM Corp., NY, USA).

## Supplementary information

Supplementary information.

## Data Availability

The data used for the current study will be available from the corresponding author upon reasonable request.

## Abbreviations

VDT: Visual display terminal
DED: Dry eye disease
OSDI: Ocular surface disease index
MGD: Meibomian gland dysfunction
TBUT: Tear fluorescein breakup time
QoL: Quality of Life
DEQS: Dry Eye-Related Quality of Life Score
SD: Standard deviation
