# Supplementary material for: Effect of warming eyelids on tear film stability and quality of life in visual display terminal users: a randomized controlled trial
Source: Sci Rep. 2020 Oct 9;10:16919. doi: 10.1038/s41598-020-73779-6 (PMC7547003; doi:10.1038/s41598-020-73779-6)
Supplement: Supplementary file 1 — Supplementary information. [file 41598_2020_73779_MOESM1_ESM.pdf]

June 17, 2015: Proposal from Kao Co.
December 15, 2015: Revised by Kao Co.

[illegible] 2015/12/18

STUDY PROTOCOL

PROTOCOL TITLE:

Evaluation of the efficacy and safety by the warming-eyelid therapy for VDT users.

PROTOCOL NUMBER: Kao-001

PROTOCOL VERSION: Version 1.1
PROTOCOL DATE: 15 Dec 2015

PRINCIPAL INVESTIGATOR:

A/Prof Ching-Hsi Hsiao, Chang Gung Memorial Hospital - Linkou, , Taiwan

STUDY SITE AND SITE PRINCIPAL INVESTIGATOR:

Country	Sites	PI
Taiwan	Chang Gung Memorial Hospital - Linkou	Ching-Hsi Hsiao

TABLE OF CONTNETS

1) Objectives 3

2) Background 3

3) Study Design 4

a) Recruitment Methods 4

b) Inclusion Criteria 4

c) Exclusion Criteria 4

d) Number of Subjects 5

e) Study Timelines 5

f) Study Endpoints 6

g) Procedures Involved in the Human Research 9

h) Study Product 11

i) Adverse Event Reporting 14

j) Withdrawal of Subjects 15

k) Protocol Deviations 15

l) Data Analysis 15

m) Sample Size and Statistical Methods 16

n) Reference 17

June 17, 2015: Proposal from Kao Co.
December 15, 2015: Revised by Kao Co.

Clinical Study Protocol

Protocol No. and Title: Kao-001

Evaluation of the efficacy and safety by the warming-eyelid therapy for VDT users.

1) Objectives:

To evaluate the efficacy and safety of the warming-eyelid therapy by the moist heat of approximately 40 degree C for 10 minutes for dry eye symptoms and symptom-induced decline of Quality of Life (QoL) in VDT users.

2) Background:

Dry eye symptom is commonly associated with ocular discomfort, ocular fatigue and irritating sensation and these symptoms are an important public health problem[1)]. According to a population-based survey of dry eye disease in the elderly (more than 65 years) in Taiwan, 33.7% is symptomatic, defined as reporting one or more frequent dry-eye symptoms[2)]. The prevalence of dry eye symptom in Taiwanese is significantly higher than in Caucasian reported based on the population-based and age-matched survey in US[3)]. Other studies have reported that dry eye disease is prevalent among young to middle-aged visual display terminal (VDT) users[4)]. It has been hypothesized that excessive evaporation of tear fluid caused by prolonged blinking intervals while gazing on VDT could be a causal factor in VDT-related dry eyes[5)]. Furthermore, these dry eye symptoms impact not only on visual performance but also on ocular and general health and well-being (general quality of life; QoL) [9)].

Warm compress has been commonly recommended as a treatment for dry eye symptom. Warming eyelids can be expected to enhance meibomian gland secretion and improved tear film stability[10)]. Heat with steam, called 'moist heat' is effective to warm the skin more widely and deeply due to the high conductivity of the moist heat than the heat without steam, called 'dry heat'[11)]. Besides, the application of the moist heat to eyes increases brain α waves and enhance parasympathetic nervous activity more than the application of the dry heat[12), 13)]. A disposable Eyelid Warming Steamer (DEWS) has been developed as a relaxing therapy for tired eyes and dry eye symptom. The DEWS is an eye mask which contains iron (Fe) and generates the moist heat by the chemical reaction of the iron with oxygen in air. The temperature of the moist heat is approximately 40 degree C and the moist heat lasts for around 10 minutes. As the DEWS begins to warm up immediately after the pouch is opened and DEWS contact with air, DEWS can be expected as an easy self-therapy to soothe dry eyes and tired eyes.

In this study, we conduct a prospective study to evaluate the effect of the warming-eyelid therapy on dry eye symptoms and dry-eye induced decreased QoL in VDT users. The efficacy and safety of the single therapy by the moist heat of approximately 40 degree C for 10 minutes are

evaluated in the single application study, and the efficacy and safety of the repeated therapy by the moist heat applied once a day for two weeks are evaluated in the repeated application study.

3) Study Design

a) Recruitment Methods

VDT-related dry eyes are involved as participants in this study. The questionnaires are provided participants to collect their information related to dry eye symptoms, VDT usage, contact lenses usage and health history and identify the potential participants. The participants' symptoms are determined by a dry eye questionnaire including twelve questions on typical dry-eye-related symptoms[14)] (Appendix A). The participants who do not usually use eye-drop will be screened preferentially, and then if the participants are not enough, the participants who usually us eye-drop will be included.

Once the potential participants who correspond to the following inclusion criteria visit our study site, the observations of ocular surface and skin are conducted. The participants who do not correspond to the following exclusion criteria take part in this study as eligible participants.

b) Inclusion Criteria

The followings are the inclusion criteria for VDT-related dry eyes (VDE);

A) Males and females aged from 20 to 69 years old

B) Participants who use VDTs, including laptops, electronic tablets, readers and smartphones for 6 hours or more a day

C) Participants who respond to more than 1 of 12 typical dry eye symptoms by "constantly" and "often" based on a dry eye questionnaire[14)].

D) Participants are able and willing to comply with all protocol requirements and procedures.

E) Participants who must be capable of providing informed consent document, with one's signature

c) Exclusion Criteria

The followings are the exclusion criteria for all participants;

A) Participants with the excessive meibomian lipid secretion (seborrheic MGD)

B) Participants with eye diseases that could affect the ocular surface (eg. Ocular inflammation, infectious conjunctivitis, allergic diseases, autoimmune diseases and collagen diseases)

C) Participants who have been treated by physicians because of eye diseases and do not recover from that disease yet at the moment of joining to this study, or participants who need to be treated by physicians because of eye disease

D) Participants with trauma, swelling and eczema at the skin around eyes

E) Participants with allergic reaction for heating, abnormality of the heat or depression of the heat

F) Participants who are deemed inappropriate to participate in this study by physicians

d) Number of Subjects

Twenty participants need to complete the research procedure with both the single and repeated application of DEWS as the eligible participants. In view of the number of participants excluding screen failures, thirty participants are expected to be pre-screened as the potential participants.

Additionally in the repeated application study, the control group with the same number of participants is also set. The total number of participants will be approximately sixty. The participants in the control group use non-warming eye mask with the same frequency as the other group, the treatment group. In terms of an ethical consideration, the control group and the treatment group are crossed over after two-week application.

e) Study Timelines

Before the single and repeated application studies are conducted, sixty participants are asked to visit the study site to take the baseline measurement. Based on the baseline data, sixty participants are randomly allocated to two groups, the treatment group.

1) In the single application study; randomized and Cross-over study

All participants are asked to visit our study site once. The participants in the treatment group use DEWS for ten minutes and the participants in the control group use non-warming eye mask for ten minutes. The same evaluations are conducted before and after the application. The duration of an individual participation is approximately one hour (i.e. the application of DEWS for ten minutes and the measurements for twenty-five minutes before and after the application).

2) In the repeated application study; randomized and Cross-over study

Participants are asked to take part in this study for four weeks and to visit the study site three times, which are 1) before the experimental period, 2) after the first two weeks and 3) after the second two weeks, and the same measurements are conducted for all participants on each visit. Participants in the treatment groups use DEWS once a day at the first two weeks and non-warming eye mask once a day at the second two weeks. Participants in the control group use non-warming eye mask once a day at the first two weeks and DEWS at the second two weeks.

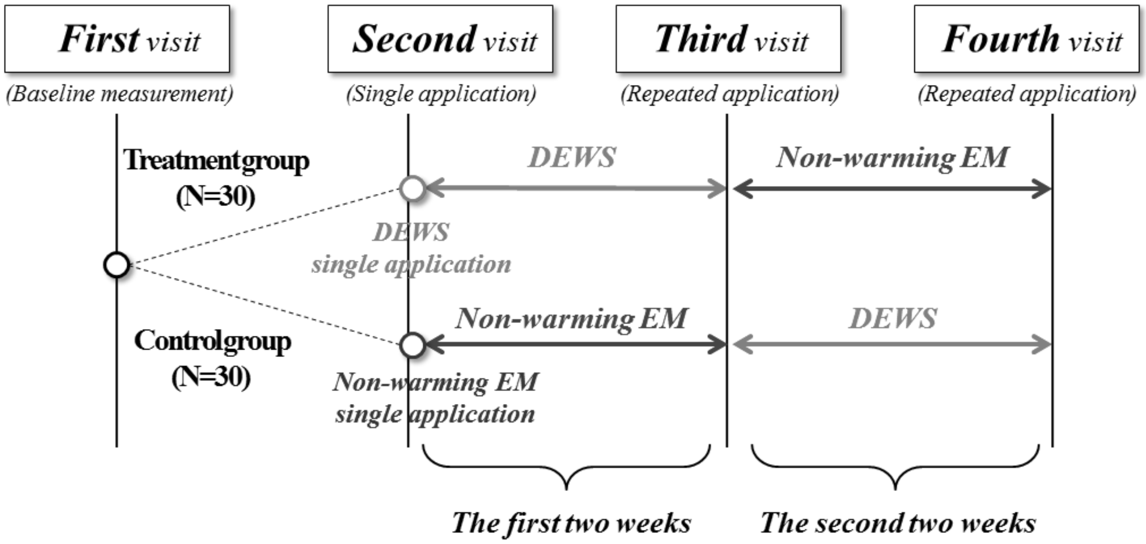

f) Study Endpoints

1) Efficacy endpoints in the single and the repeated application study

The identical endpoints to evaluate the efficacy of DEWS are carried out in both the single application study and the repeated application study.

The primary endpoint is judged from the assessment for the severity of the symptoms subjectively using visual analog scale (VAS) and the symptom-related quality of life using Dry Eye-Related Quality of Life Score (DEQS) [15]). The secondary endpoints are judged from the other objective measurements.

- ***Assessment of symptoms and mood state evaluated by participants (Appendix B)***

  The severity of symptoms and mood state are evaluated using a VAS by each participant. A VAS is a horizontal line, 100mm in length, anchored by word descriptors at each end. Participants mark on the line the point that they feel their perception of their current state. The VAS score is determined by measuring the length from the left hand end of the line to the point that they mark. The five symptoms to be evaluated are: tiredness of eyes, dryness of eyes, grittiness of eyes, soreness of eyes, irritating sensation of eyes and ocular discomfort. Each symptom is relieved as the VAS score decreases. The mood states to be evaluated are: relaxation, comfort and refreshed feeling. Each mood state is enhanced as the VAS score increases.

- ***Assessment of the quality of life (DEQS)[15]) evaluated by participants (Appendix C)***

  Dry eye related quality of life is assessed using the dry eye-related quality of life score questionnaire, DEQS. The DEQS can assess various aspects of Quality of Life including its mental aspect. This questionnaire consists of 15 items related to dry eye symptoms and

influence on daily life, and the overall degree of QoL impairment is calculated as a summary score (0 to 100). The DEQS score is decreased as QoL is improved.

- ***Measurement of tear lipid layer thickness using tear interference camera (DR-1) or Oculus Keratograph by an examiner***

Superficial tear lipid layer is observed with tear interference camera (DR-1). The participant is instructed to blink naturally and then the interference images are monitored. After a few seconds of blinking, when the interference image becomes stable, the image is captured. The interference images are semi-quantitatively graded on the pattern and color[18)] and tear lipid layer thickness is quantified with the color chart system[19)]. Meibography could be taken at the same time by Oculus Keratograph.

- ***Tear film breakup time (TBUT) evaluated by an examiner and/or Oculus Keratograph***

TBUT is the parameter of tear film stability, defined as the time to initial breakup of the tear film after a blink[16)]. The liquid of fluorescein is instilled by the micropipette to stain the ocular surface. After instillation, the participant is asked to blink several times and to move the eyes, to mix the fluorescein in the tears. Observation is conducted with the slit lamp. The participant is instructed to blink naturally and then, once homogeneous tear film fluorescence is confirmed, he/she is asked to keep eyes open while looking straight ahead. The time from upstroke of the last blink to the first tear film break is recorded as the TBUT measurement. The tear film stability is improved as TBUT increases. The average of three consecutive trials is the final value. The results are recorded in a case report form.

- ***Staining of ocular surface evaluated by an examiner***

Ocular surface damage is assessed by staining cornea and conjunctiva. The liquid of fluorescein is instilled by the micropipette to stain the ocular surface. After instillation, the participant is asked to blink several times and to move the eyes, to mix the fluorescein in the tears. Observation is conducted through a yellow barrier filter to assess both the cornea and conjunctiva together. The staining of ocular surface is graded 0 to 3 on the cornea and two exposed conjunctival segments (range: 0-9) according to the van Bijsterveld system[17)]. The staining score is recorded in a case report form.

- ***Schirmer test evaluated by an examiner***

Schirmer test is an estimation of tear flow reflex by insertion of a filter paper into the conjunctival sac[17)]. A Schirmer paper strip is inserted over the lower lid margin, midway between the middle and outer third. This test is performed with the participants' eyes closed in 5 minutes. The length of wetting after 5 minutes is recorded as the Schirmer test score in a case report form.

- ***Meibomian gland expression evaluated by an examiner***

Meibomian gland expression can be performed as an indicator of meibomian gland function, meibum expressibility and quality. The secretion expressed from the glands is observed with a physical force applied to the outer surface of the eyelid to determine whether a specific meibomian gland is functional and capable of providing secretion. The meibum expressibility and quality are graded by four-point scales defined for meibum expressibility; 0=clear meibum, easily expressed, 1=cloudy meibum, easily expressed, 2=cloudy meibum expressed with moderate pressure and 3=meibum not expressible, even with hard pressure[20)] and for meibum quality; 0=clear fluid, 1=cloudy fluid, 2=cloudy particulate fluid and 3=inspissated, like toothpaste[21)]. The meibum secretion is improved as the scores of meibum expressibility and quality decreases. The results are recorded in a case report form.

2) Safety endpoints in the single application study

The endpoints for the ophthalmologic safety are evaluated by visual acuity, intraocular pressure and ocular surface/eyelids findings.

− ***Visual acuity evaluated by an examiner***

The normal visual acuity is measured for both eyes separately using a Snellen chart. An ophthalmologist carries out a single measurement for each eye and confirms whether there is not difference between the value before and after the application of DEWS. The results are recorded in a case report form.

− ***Intraocular pressure evaluated by an examiner***

The intraocular pressure is measured for both eyes separately using the tonometer. An ophthalmologist carries out a single measurement for each eye and confirms whether there is not difference between the value before and after the application of DEWS. The results are recorded in a case report form.

− ***Ocular surface/eyelids findings diagnosed by an examiner***

An ophthalmologist observes the cornea, conjunctiva and eyelids for each participant and confirms whether there is any inflammation and hyperemia on the ocular surface or any other abnormalities on the ocular surface and eyelids before and after the application of DEWS. The results are reported in a case report form.

3) Safety endpoints in the repeated application study

In the repeated application study, a dairy is completed by a participant in addition to the identical endpoints for the safety evaluation in the single application study.

− ***Diary reported by a participant (Appendix D)***

Participants are asked to inscroll the time, the date, the place and adverse events, if there is, in a daily questionnaire after every use of DEWS. If there is any report of adverse events, an ophthalmologist determines the severity and the relationship of the adverse events to using DEWS.

The following table is showing the list of the examinations in this study. X in each cell means we will conduct these examinations in each visit and the number in each cell is the order in which the examinations are conducted.

**List of examinations**<br>*Note:*<br>These number in the cell is indicating the order of the examinations to be done in each visit.	*Screening*		*Treatment phase*			
		First visit	Second visit		Third visit	Fourth visit
		Day -4 ~ Day -2	Day 1		Day 15	Day 29
	Pre-screening	Screening	Single application		Repeated application	Repeated application
			Pre (baseline)	Post		
1) Dry eye symptom related to VDT work<br>- Dry eye questionnaire<br>**[Appendix A]**	X_2				X_1	X_1
2) Informed consent	X_1					
3) Skin type assessment<br>- Fitzpatrick skin type<br>**[Appendix E]**		X_3				
4) Dry eye symptom<br>- Visual analog scale (VAS)<br>**[Appendix B]**			X_1	X_7	X_2	X_2
5) Quality of life related to dry eye symptoms<br>- Dry eye related quality of life score (DEQS)<br>**[Appendix C]**			X_2		X_3	X_3
6) Visual acuity<br>- Snellen chart			X_3	X_1	X_4	X_4
7) Intraocular pressure<br>- Tonometer			X_4	X_2	X_5	X_5
8) Lipid layer thickness<br>- DR-1 or Oculus			X_5	X_3	X_6	X_6
9) Ocular surface stability<br>- Tear film breakup time by an examiner and/or Oculus			X_6	X_4	X_7	X_7
10) Ocular surface damage<br>- Staining of ocular surface			X_7	X_5	X_8	X_8
11) Tear flow reflex<br>- Schirmer test		X_5			X_10	X_10
12) Meibum expressibility/quality<br>- Meibomial gland expression test		X_6		X_7	X_11	X_11
13) Ocular surface/eyelid findings<br>- Findings by a physician		X_4	X_8	X_6	X_9	X_9
14) Diary reported by participants<br>- Daily report<br>**[Appendix D]**					X	X

Table. The list of the examinations to be conducted in each visit. 'X' and the number in the next to 'X' indicate the examinations which need to be conducted in the visit and the order of the examinations, respectively.

g) Procedures Involved in the Human Research

0) The First visit: Screening of the eligible participants with signed and dated written <u>Informed consent</u>

The potential participants who respond to more than 1 of 12 typical dry-eye-related symptoms by “constantly” or “often” based on the dry eye questionnaire is are asked to visit our study site. The participants who usually wear contact lenses are asked to take them off before visiting study site. At this visit, the investigators fully explain the nature of the procedures of the study for each participant at first. A participant is then enrolled with written informed consent. They are asked to complete the personal information questionnaire including the questionnaire on the skin phototype questionnaire (appendix E) [22).

After that, the ocular surface findings and the skin around eyes findings, Schirmer test for their left eye and meibomian gland expression test are conducted by an examiner in this order. The participants who correspond to the inclusion criteria and who do not correspond to the exclusion criteria are involved in this study as the eligible participants. All examinations are carried out for their both eyes.

Eye-drop users are asked to try not to use the eye-drop throughout this study. If they bear the eye dryness, they are allowed to use the eye-drop with writing down the usage of the eye-drop in the daily report.

1) The Second Visit: Baseline collecting and Single application study

Once the eligible participants (describe “the participants” from here) visit our study site, they are asked to complete VAS on their symptoms and mood state at the moment on that day and DEQS in the last week, and then the measurements of visual acuity and intraocular pressure are carried out. After that, the lipid layer thickness measurement, TBUT measurement, the ocular surface findings by staining, the eyelids findings by an examiner are carried out in this order. All examinations are carried out for their both eyes.

After that, the participants use DEWS for 10 minutes in the sitting posture. Investigators measure the time and tell participants after 10 minutes application.

After 10 minutes application, the measurements of visual acuity and intraocular pressure are carried out. After that, the lipid layer thickness measurement, TBUT measurement, the ocular surface and the eyelids findings, the assessment of the meibomian gland expression by an examiner are carried out in this order. After finishing these objective measurements, the participants complete VAS assessment on their symptoms and mood state at the moment. All examinations are carried out for their both eyes.

For the management of the risk for participants, the use of DEWS is only at the predetermined location with an examiner’s or an investigator’s presence. During the application, investigators ask participants the warmth. If the participants feel something wrong with DEWS by any chance, they should take it off immediately. It should be reported in derail in a case report form.

2) The Third Visit and The Fourth Visit: The repeated application study

The participants in the treatment group are asked to use DEWS once a day for the first two weeks and then use non-warming eye mask one a day for the second two weeks. The participants in the control group are asked to use non warming eye mask once a day for the first two weeks and then DEWS once a day for the second two weeks. All participants are asked to visit our study site after the first two weeks (the third visit) and also after the second two weeks (the fourth visit). The participants who usually wear contact lenses are asked to take them off before visiting study site on both days.

At the third visit and fourth visit, the participants complete Dry eye questionnaire, VAS on their symptoms and mood state at the moment on that day and DEQS in the last week, and then the measurements of visual acuity and intraocular pressure are carried out by an examiner. After that, the lipid layer thickness measurement, TBUT measurement, the ocular surface and the eyelids findings, Schirmer test, and the assessment of meibomian gland expression by an examiner are carried out in this order. All examinations are carried out for their both eyes.

h) Study Product

1. The study product

The study product, the disposable Eyelid Warming Steamer (DEWS), is an eye mask which contains iron (Fe) and generates the heat with the steam (the moist heat) by the oxidative reaction of the iron with oxygen in air. DEWS is packed in the pouch, and once the pouch is opened, DEWS begins to warm up immediately with the oxidative reaction initiated. The temperature of the moist heat is approximately 40 degree C and the moist heat lasts for around 10 minutes. The temperature of DEWS is designed to keep the participants away from getting burned, therefore, when DEWS applied to the eyelids, the eyelids skin temperatures do not exceed up to 43 degree C and the duration of the temperature over 40 degree C do not exceed up to 15 minutes. DEWS has ear straps and the participants use DEWS with the ear straps placed around their ears. Non-warming eye mask is prepared for a control of DEWS as it does not generate the heat and the steam. The appearance of the non-warming eye mask and its package is totally the same as DEWS.

2. Names of the Study Products

(1) **Study Product: Disposable Eyelid Warming Steamer (DEWS)**

Route of administration: for external use, once daily

Dose(s): 1 mask

Dosing schedule: a participant needs to use DEWS once a day for 14 days, and each DEWS needs to be applied at least for ten minutes.

(2) **Control Product: non-warming eye mask**

Route of administration: for external use, once daily

Dose(s): 1 mask

Dosing schedule: a participant needs to use non-warming eye mask once a day for 14 days, and each non-warming eye mask needs to be applied at least for ten minutes.

3. Usage directions

In the repeated application study, the study product must be used on the participants' own. The participants must thoroughly read the usage directions provided by investigators before using and use the study product with keeping it in mind. The participants who are usually wearing contact lenses are required to use the study product without wearing the contact lenses.

The study product is packed in a silver pouch. Participants open the pouch and remove the study product from the pouch. And then, they tear along perforation and place ear straps around ears. The study product begins to warm up as soon as pouch is opened, therefore participants must use the study product immediately after the pouch is opened.

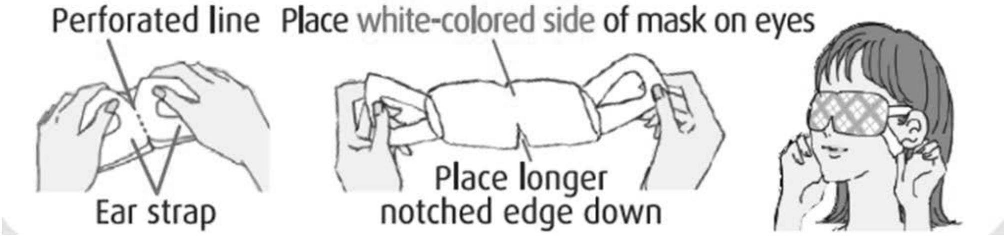

While using the study product, participants should keep eyes closed. If eye drops are applied, please wait for a few minutes before using eye mask.

There is a number from 01 to 14 on the pouch. The participants should use the study products in this order so that the investigators could trace which study product had been used on each day after this study.

4. Packaging and Labeling of the Study Products

Study products will be packed individually in silver pouches for each usage. Each silver pouch will contain one mask. The silver pouch packed for the use period is illustrated and each package will be labeled according to the local requirements.

Appearance of study products

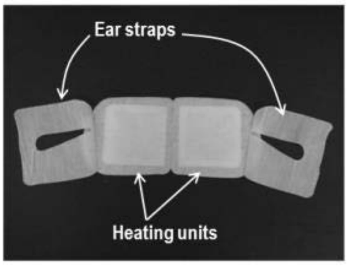

White-colored side to be applied to skin around eyelids

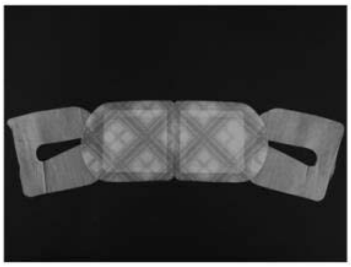

Purple-printed side facing to outside, NOT to be applied to skin

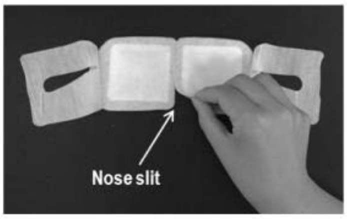

Longer notched edge to be placed down

Appearance of silver pouch

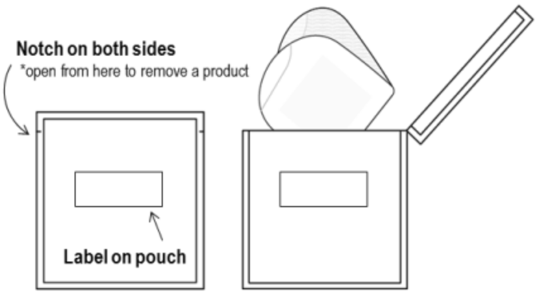

A study product packed in a silver pouch

Appearance of zipper bag

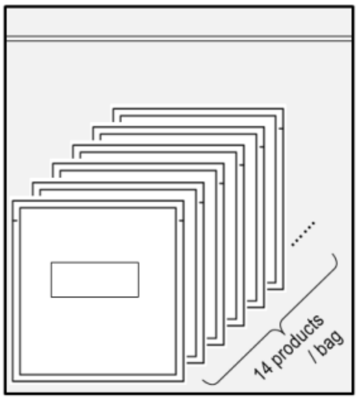

14 study products for 2-week usage in a plastic zipper bag

5. Storage Conditions

The study product must be kept away from the direct sunlight, high temperature and heat sources (heaters etc.). The study product may not heat up if the pouch is scratched. The study product which was already used by participants must be put back in the original pouch and kept away from the study product to be used.

6. Methods of Handling, Storage, and Management Accountability of the Study Products

The study products, is manufactured and supplied by Kao Corporation, Japan. It must be kept in a secure area and should be kept away from the direct sunlight, high temperature and

heat sources (heaters etc.). All study products will be used around the eyelid area by a participant and dispensing and accounting by study staff in the study site.

During the using period, if an unused mask is missing to use, a participant must report the missing in the daily report provided and then they must report the missing to the investigator at the visit to the study site. The study nurse must report their missing in a case report form as a protocol deviation.

All study products and all control products must be return to the investigator regardless of whether they were used or not used by participants. So the both of product which was already used by participants must be put back in the original pouch and kept away from the study product to be used by participants until their next visit.

i) Adverse Event Reporting

During each two weeks, participants are asked to inscroll the time, the date, the place and adverse events if any in a daily report after using DEWS or non-warming eye mask. Participants are required to suspend using DEWS or non-warming eye mask immediately and report the details to the principal investigator immediately when any abnormality or any adverse event on their skin or their ocular surface is occurred. In that case, an ophthalmologist examines the ocular surface and/or the eyelids and determines the severity and the relationship to using DEWS or non-warming eye mask. All these cases are reported in a case report form.

Action to Be Taken in the Event of Serious Adverse Events

If an SAE occurs during the study period, the investigator (or sub-investigator) should immediately apply appropriate treatment for the SAE to subject regardless of whether or not the event is related to the study product. The investigator (or sub-investigator) should report any SAE to CRO by fax using an ADR form within 24 hours after the onset of SAE.

The investigator will report details of the relevant SAE in writing to the CRO within 7 days after the first report to the CRO. The investigator will also report relevant safety information to regulatory authorities and IRBs/IECs within the specified timeframe in accordance with the local regulations and laws of country.

[Definition of an SAE]

1. Results in death,
2. Is life-threatening
3. Requires inpatient hospitalization or prolongation of the existing hospitalization,
4. Results in disability,
5. May result in disability,
6. Is considered serious with reference to the above 1 to 5, or
7. Is a congenital anomaly/birth defect

During the study, if a participant has a hospitalization or procedure that was scheduled before the study entry, ie, before informed consent for an event/condition that occurred before the study, the hospitalization is considered a therapeutic intervention and not the result of a SAE. However, if the event/condition worsens during the study, it should be reported as an AE.

j) Withdrawal of Subjects

Participants are withdrawn from this study at the following cases.

1) In the case that a participant lodges to withdraw after the informed consent is obtained
2) In the case that the principal investigator deems to need to cease their sustained participation due to any adverse event during this study
3) In the case that the principal investigator deems inappropriate to participate due to any health problem after the informed consent is obtained

The reason for a participant's discontinuation to use DEWS will be documented in the end of study/withdrawal CRF.

k) Protocol Deviations

The investigator (or sub-investigator) should document any deviations from the protocol regardless of their reasons. Only when the protocol was followed in order to avoid an immediate hazard to study participant or for other medically compelling reason, the investigator should prepare and submit the records explaining the reason thereof to the sponsor/CRO, and retain a copy of the records.

l) Data Analysis

1) Statistical Analysis

(1) Continuous Variable

The raw data will be summarized as the number of observations, mean, median, standard deviation, minimum and maximum on each-visit basis. Normal distributed data will be analyzed using t-test or ANOVA. The Wilcoxon Rank Sum test or Mann-Whitney's test will be adopted when the hypothesis of normal distribution is violated.

(2) Categorical Variable

The raw data will be summarized as the number of observations, frequency of each class on each-visit basis. For nominal variables, chi-square test or Fisher's exact test will be adopted as needed. Ordinal variables will be analyzed using Cochran-Mantel-Haenszel (CMH) test.

2) Analysis Population

Data analyses and summaries of the efficacy and safety endpoints will be performed in the following populations:

Per-protocol (PP) population --- all randomized participants who take the study treatment and who complete the study without any major protocol deviation.

I**ntention to Treat (ITT) Population ---** all randomized participants who have taken the study treatment and have at least one follow-up evaluation for primary efficacy endpoint, regardless of their compliance with the protocol or their eligibility to the study.

Safety Population --- participants who have taken at least one dose of the study treatments.

The primary efficacy evaluation will be performed on both ITT and PP population. The secondary efficacy evaluation will be performed on the ITT population. Safety endpoints will be analyzed using the safety population.

m) Sample Size and Statistical Methods

1) **Sample size**

Approximately 60 participants will be screened and enrolled to obtain a minimum of 20 evaluable participants per group.

2) **Assessment of Efficacy**

Efficacy endpoints will be listed and summarized as appropriate: mean/median, standard deviation, minimum, maximum and total numbers for normally distributed data; median and range for non-normally distributed data; frequencies, total numbers and percentages for categorical data.

The demographical characteristics in both treatment and control group will be compared at baseline with chi-square test for categorical variables and t-test for continuous variables.

(1) <u>Primary Efficacy Endpoint:</u>

a. The severity of symptoms and the mood state using VAS and increase of VAS will be evaluated by two sample t-tests or ANOVA.

b. The dry eye related quality of life score (DEQS) and change of DEQS will be evaluated by chi-square test, Fisher's exact test or appropriate non-parametric method.

(2) <u>Secondary Efficacy Endpoint:</u>

a. Ocular surface stability (tear film breakup time, TBUT) and increase of TBUT will be evaluated by two sample t-tests or ANOVA.

b. Shirmer test value (tear flow reflex) and change of Shirmer test value will be evaluated by two sample t-tests or ANOVA.

c. The staining score (Ocular surface damage) and change of staining score will be evaluated by chi-square test, Fisher's exact test or appropriate non-parametric method.

d. The thickness (tear film thickness) and change of thickness will be evaluated by two sample t-tests or ANOVA.

e. The expressibility score (meibum expressibility) and change of score will be evaluated by chi-square test, Fisher's exact test or appropriate non-parametric method.

f. The quality score (meibum quality) and change of score will be evaluated by chi-square test, Fisher's exact test or appropriate non-parametric method.

3) **Assessment of Safety**

(1) The visual acuity (Snellen chart) using log MAR will be evaluated by two sample t-tests or ANOVA.

(2) The tonometer (intra ocular pressure) using IOP will be evaluated by two sample t-tests or ANOVA.

(3) Adverse events will be tabulated, and the incidence rates will be calculated.

n) Reference

1) Miljanovic B et al., Impact of dry eye syndrome on vision-related quality of life. *Am J Ophthalmol* 143 (3): 409-415, 2007.

2) Lin PY et al., Prevalence of dry eye among an elderly Chinese population in Taiwan: the Shihpai Eye Study. *Ophthalmology* 110: 1096-1101, 2003.

3) Schein OD et al., Prevalence of dry eye among the elderly: a population-based assessment. *Arch Intern Med.* 159: 1359-1363, 1999.

4) Uchino M et al., Prevalence of dry eye disease and its risk factors in visual display terminal users: The Osaka Study. *Am J Ophthalmol* 156 (4): 759-766, 2013.

5) Tsubota K et al., Effect of ocular surface area and blink rate on tear film dynamics. *Arch Ophthalmol.* 113(2): 155-158, 1995.

6) Shtein RM, Post-LASIK dry eye. *Expert Rev Ophthalmol.* 6(5): 575-582, 2011.

7) Albietz JM et al., Dry eye after LASIK: Comparison of outcomes for Asian and Caucasian eyes. *Clin Exp Optom.* 88(2): 89-96, 2005.

8) Li XM et al., Investigation of dry eye disease and analysis of the pathologic factors in patients after cataract surgery. *Cornea* 26: 16-20, 2007.

9) DEWS, The epidemiology of dry eye disease: Report of the epidemiology subcommittee of the international Dry Eye Workshop. *Ocul Surf.* 5(2): 93-107, 2007.

10) Geerling G et al., The international workshop on meibomian gland dysfunction: Report of the subcommittee on management and treatment of meibomian gland dysfunction. *Invest Ophthalmol Vis Sci* 52: 2050-2064, 2011.

11) Abramason Di et al., Comparison of wet and dry heat in raising temperature of tissues. *Arch Phys Med Rehabil* 48: 654-661, 1961.

12) Ochiai R., Moist heat stimulation influence on electroencephalograms and the autonomic nervous system [in Japanese]. *Jpn J Auton Nervous Syst*, 38: 450-454, 2001.

13) Nagashima Y., Effect of autonomic nervous activity of the application of heat- and steam-generating sheets to the eyes [in Japanese]. *Jpn J Auton Nervous Syst*, 43 260-268, 2006.

14) Toda I et al., Ocular fatigue is the major symptom of dry eye. *Acta Opthalmol (Copenh)* 199; 71(3): 347-352.

15) Sakane Y et al., Developent and validation of the dry eye-related quality of life score questionnaire. *JAMA Ophthalmol* 131 (10): 1331-1338, 2013.

16) DEWS, The definition and classification of dry eye disease: Report of the definition and classification subcommittee of the international Dry Eye Workshop. *Ocul Surf.* 5(2): 75-92, 2007.

17) DEWS, Methodologies to diagnose and monitor dry eye disease: Report of the diagnostic methodology subcommittee of the international Dry Eye Workshop. *Ocul Surf.* 5(2): 108-152, 2007.

18) Yokoi N et al., Correlation of tear lipid layer interference patterns with the diagnosis and severity of dry eye. *Am J Ophthalmol.* 122: 818-824, 1996.

19) Goto E et al., Computer-synthesis of an interference color chart of human tear lipid layer by a colorimetric approach. *Invest Ophthalmol Vis Sci.* 44: 4693-4697, 2003.

20) Shimazaki J et al., Ocular surface changes and discomfort in patients with meibomian gland dysfunction. *Arch Ophthalmol.* 113: 1266-1270, 1995.

21) Bron AJ et al., Meibomian gland disease. Classification and grading of lid changes. *Eye.* 5: 395-411, 1991.

22) Thomas BF et al., The Validity and Practicality of Sun-Reactive Skin Types I through VI., *Arch Dermatol.*, 124(6): 869-871, 1988.
